# Guidelines and recommendations for radiologist staffing, education and training

**DOI:** 10.1186/s13244-025-01926-6

**Published:** 2025-03-12

**Authors:** Adrian P. Brady, Christian Loewe, Boris Brkljacic, Graciano Paulo, Martina Szucsich, Monika Hierath, on behalf of the European Society of Radiology

**Affiliations:** 1 Department of Radiology, University College Cork, Cork, Ireland; 2 Division of Cardiovascular and Interventional Radiology, Department of Biomedical Imaging and Image-Guided Therapy, Medical University of Vienna, Vienna, Austria; 3 Department of Diagnostic and Interventional Radiology, University Hospital Dubrava, University of Zagreb School of Medicine, Zagreb, Croatia; 4 Polytechnic University of Coimbra, IPC-ESTESC - Coimbra Health School, Medical Imaging and Radiotherapy, Coimbra, Portugal; 5 H&TRC- Centro de Investigação em Saúde e Tecnologia, Lisbon, Portugal; 6 European Society of Radiology, Vienna, Austria

**Keywords:** Radiology, Workforce, Radiation protection, Education and training, Basic Safety Standards Directive

## Abstract

**Abstract:**

This article outlines the radiology-related staffing and education/training guidelines and recommendations developed by the European Commission-funded EU-REST (European Union Radiation, Education, Staffing & Training) project. The radiologist consortium partners propose the use of hour of machine/system/activity as the basic unit to calculate radiologist staffing needs. Education and training recommendations for radiologists include establishing 5 years as the standard duration of specialty training in radiology and establishing the ESR European Training Curriculum for Radiology as the European-wide standard. General recommendations for all professional groups include the maintenance of a central registry for each professional group and for relevant equipment, by each EU Member State, mandated CPD including techniques and knowledge relevant to each professional group, adoption vs adaptation of the project’s recommendations.

**Critical relevance statement:**

The radiology-related staffing and education/training guidelines and recommendations developed by the EU-REST project propose a novel approach to calculate radiologist staffing numbers and provide recommendations regarding radiology education and training as well as general recommendations for all professional groups covered by the project.

**Key Points:**

- The pros and cons of taking population, workload, equipment or bed availability numbers as parameters to calculate radiology workforce are described.
- The reasons why these parameters are not suitable to calculate radiologist staffing needs are explained.
- The proposed use of hour of machine/system/activity as the basic unit to calculate radiologist staffing needs allows for establishing an adaptable and scalable guideline.
- Education and training recommendations for radiologists and non-profession-specific recommendations are summarised.

## Introduction

In our previous two articles, we reported on the origin, design and conduct of the EU-REST project [1, 2] and on the information gathered relating to the current status of radiologist workforce availability, and to education and training in radiology in the 27 EU Member States [3]. The EU-REST project was commissioned and designed by the European Commission (EC) to characterise the current status for these parameters and, crucially, to develop guidelines and recommendations for EU Member States with respect to radiologist workforce needs, and the educational and training requirements for radiologists. The project was funded by the EU4Health Programme of the EU [4]; it was intended to form part of the actions of the Strategic Agenda for Medical Ionising Radiation Applications (SAMIRA) Action Plan and to contribute to the implementation of Europe’s Beating Cancer Plan [5].

In this, third article, we report on the guidelines and recommendations developed during the project, submitted to the EC, and accepted by the Commission as part of the final project report.

The EU-REST project encompassed all professional groups involved in medical applications of ionising radiation, and the other groups who formed part of the project consortium (Radiation Oncologists, Nuclear Medicine Physicians, Radiographers, Medical Physicists/Medical Physics Experts and Radiation Therapists) will also report separately on the project and its outcomes, as they relate specifically to their professional groups.

Some of the recommendations that emerged from the project relate to all involved professions and will also form part of this article.

## General principles

The guidelines developed as part of the EU-REST project were based on the following 3 pillars:

i. Existing practice across the 27 EU Member States
  Each professional group aimed to identify consistencies/uniformity in current reasonably good practice from the data available, i.e., the results of the EU-REST survey (as reported for radiologists in Part 2, EU-REST—current status of radiologist staffing, education & training in the 27 EU Member States [3]) and the literature review.
  A general lack of generally applicable literature in this area was acknowledged; whenever possible these guidelines were established based on evidence-based research and evidence-based practice.
ii. Recommendations
  Any recommendations were made to influence correct practice while reflecting minimum requirements.
  Any recommendations are supported by authoritative literature, guidelines, evidence-based research, or consensus papers, where available. However, it must be stressed that the project literature survey demonstrated a significant deficit in existing literature defining appropriate standards for the matters in question, especially with respect to appropriate radiologist staffing numbers.
iii. Improvements
  Any changes or improvements which are obvious and required, are supported by data from i. and ii. above and agreed by all consortium partners. These predominantly constituted the general guidelines, applicable to all professional groups, as outlined below.

## Staffing guidelines for radiologists

Guidelines were developed to consider the level of relevant imaging equipment available, expected workload, and the complexities of practices undertaken. Irrespective of size of department or complexity of work, an essential methodology to calculate the minimum number of radiologist staff for each modality was defined as a starting point, with additional staffing requirements defined using the presented methods, based on increasing complexity of work, workload, equipment levels and the introduction of new roles and responsibilities. The aim was to provide guidelines on methods of calculating staffing needs, both for current practice but also for a future expansion of services or new roles. This will ensure long-term applicability of the project outputs.

Before defining the staffing guidelines which formed one of the key outputs of the project, consortium members considered methods which have been used in the past to measure radiologists’ work.

Measuring how much work is done by a radiologist is a far-from-simple task. Many efforts have been made in the past to define reproducible, accurate and scalable methods, with little or no success in achieving widespread acceptance. Among these methods have been:

1. Crude study numbers [6]. Somewhere between 10,000 and 20,000 procedure reports per annum was used as a benchmark for appropriate annual activity for an individual radiologist. This had some validity when radiology activity was mostly based on plain radiography but became meaningless once more-complex imaging modalities became commonplace. The amount of time required to report a plain radiograph (often < 1 min) bore no relationship to the time for a multiphasic CT or a multiparametric MR (potentially up to 1 h) and counting these activities as representing the same output was not reasonable. An Irish National Workload review in 2011 described this method as “old-fashioned, discredited and [an] inappropriate misuse of data” and stated that such data “should not be used in an unfiltered and un-weighted manner” [7].
2. As cross-sectional imaging utilisation grew, attempts were made to stratify radiologists’ activities depending on the modalities they were reporting, including recommendations issued in 1999 by the Royal College of Radiologists in the UK [8]. This document suggested an appropriate workload for a notional half-day, varying according to the modality involved. However, it did not allow for radiologist activity that could not be measured in number of reports generated, such as multidisciplinary team (MDT) activity, procedural work, teaching, research, administration, etc.
3. Relative Value Unit (RVU) measurements were developed in a number of countries (including the USA, Canada, Australia and New Zealand) in an effort to overcome some of the difficulties with earlier measurement methods [9, 10]. Some (but not all) of these systems incorporated attempts to measure both technical and procedural elements of radiologist workload, making allowance for the time required, the complexity and the intensity of specific pieces of work, but all suffered from being primarily designed and used to determine reimbursement for work done, rather than to measure individual workload. RVUs focused purely on reporting time and took no account of other aspects of a radiologist’s work.
4. Later methodologies were developed by academic bodies in some countries to make allowance for the non-reporting elements of a modern radiologist’s work (as mentioned in item 2 above) [6, 11, 12]. These had limited local success in the countries in which they were developed in terms of helping to redefine the scope of work of a radiologist, but had little overall impact on radiologist numbers and no international penetration as general standards.

Evaluating all these methodologies, one cannot help but think of the tongue-twister “How much wood could a woodchuck chuck if a woodchuck could chuck wood?”. Despite the fact that all these ideas mentioned above may each have seemed relevant and appropriate at the time, it became obvious that the wrong question was being asked, in an effort to provide a numerical answer that was unattainable, not representative of real-world radiological practice, and therefore doomed to failure.

A fundamental point that must be grasped is that no generally accepted/agreed definitions exist for the

i. Number of examinations needed per population,
ii. Number of pieces of equipment needed per population,
iii. Appropriate per-radiologist reporting output.

In recent years, fortunately, awareness has grown of a number of significant pertinent aspects of considering radiology workload and radiology’s impact on healthcare and well-being. First, understanding has grown that the role of the radiologist goes far beyond the production of reports of imaging studies or the performance of interventional procedures [13], despite these being key components of radiologists’ work. In particular, the intensification of the involvement of radiologists in multidisciplinary care of patients has emphasised radiologists’ clinical input and role in promoting health and well-being and optimising outcomes for patients. Second, the value-based radiology concept and movement is increasingly focusing on defining the value provided to patients on an individual basis and to society in general rather than assessing radiology’s contribution based on activity volume alone [14–17].

Bearing all of this in mind, any guideline for appropriate workforce in radiology recommended as an outcome of the EU-REST project needed to incorporate the following elements:

1. Non-countable (by numbers of reports or other outputs) activity (e.g., MDT work, direct patient engagement, etc.) must be provided for and appropriately recognised as valid and valuable. Indeed, a recent publication from the Netherlands confirmed that employers of radiologists in that country are increasingly seeking applicants with teaching, research, and management skills, in addition to their clinical competencies [18].
2. Value contributions to individual patients and society in general must be considered.
3. If available, existing and working guidelines from the EU 27 countries should be included and/or adapted. Unfortunately, data collection as part of the EU-REST survey identified no such usable existing guideline. There is no uniform method used across a range of countries to determine workforce numbers in radiology. Many countries use local, bespoke methods, or have no specific method at all. In some instances, workforce provision is determined by “market forces”: how many radiologists need to be hired to deal with the workload presented (and, sometimes, to optimise earnings for departments and radiologists individually), balanced in some way by how many trainees are produced each year, or how many potential employees are available.
4. If available, recommendations supported by authoritative literature/guidelines/research, etc., should be incorporated and/or adapted. Again, unfortunately, data collection as part of the EU-REST review of existing literature identified no usable existing guideline.
5. Any guideline recommended should be adaptable at least for the short-to-medium term future. Medical and radiological practice is constantly in flux, as patterns of utilisation of investigative methods change and new tools become available. Adopting a guideline today which is outdated in 5 years is of little overall value.
6. Any guideline recommended should be adaptable for differences in practice and imaging availability between countries, and should be scalable, such that the guideline can be applied on a local, regional, national, or multi-national basis.

Therefore, given the unavailability of any existing guideline or workforce determination method which could fulfil the needs outlined above, we were faced with the need to propose a “new” method of determining workforce needs, based on calculable denominators which can be generalised across many countries and practice styles.

### Possible approaches to estimate/calculate workforce numbers

For radiologists, we considered several possible methods for calculating appropriate workforce numbers in some locations, each with pros and cons.

#### Based on population

At first glance, basing guidance on the number of radiologists needed to service a given population on that population’s size seems intuitive. For that reason, we will explore this option in particular detail. If a population increases or decreases over time, it would seem sensible to plan to change radiologist numbers to match such changes. However, this is a very crude measure and has many disadvantages.

One could attempt to adapt a population-based formula, to take account of the specific population demographics (age profile, etc.), but this would not overcome some of the other difficulties with such a crude measurement system (Table 1).

**Table 1:** Pros and cons: basing radiologist numbers on population

Pros	Cons
Applicable to all countries	Ignores age-profile demographics (young or old population, etc.)
Avoids issues relating to public/private practice	Ignores variation in complexity between countries
Relatively simple to calculate	Slow to change, and assumes work practices do not change with time (independently of population)
	Frequency of measuring population size (censuses) makes it difficult to adapt radiologist numbers quickly
	Numbers of radiologist training positions needed to meet population-based standards will always lag substantially behind actual population (given a minimum lead time for training new radiologists of at least 5 years)

OECD and WHO public reports primarily present data on healthcare in general and on the healthcare workforce (HCW) in different countries based on population calculations [19, 20]. With respect to workforce, the most commonly used indicator is the density of the healthcare workforce (i.e., the number of active HCWs in an occupation divided by the population). The workforce density, usually presented per 10,000 people, is a simple measure useful for the basic comparisons between countries and different healthcare occupations. It is applicable to all countries and avoids issues relating to public/private practice. Thus, a seemingly logical step in drafting guidelines for appropriate numbers of radiologists would be to specifically calculate desirable numbers of radiologists based on the population of the country/region they are serving.

The latest OECD report demonstrates that in OECD member countries, health and social care systems employ more workers now than at any other time in history. In 2019 one in every ten jobs (10%) was in health or social care, up from less than 9% in 2000. In Nordic countries and the Netherlands, more than 15% of all jobs are in health and social work. On average across OECD countries, employment in health and social work increased by 49% between 2000 and 2019, outpacing all other sectors, even the service sector. In OECD countries the number of doctors increased considerably, from around 2.8 million in 2000 to 4.1 million in 2019 (an increase from 2.7 per 1000 population in 2000 to 3.6 per 1000 in 2019). Despite this overall trend, differences in doctor density across OECD European countries are large: Poland and Turkey have 2.5 doctors per 1000 population, while Austria, Portugal, and Greece have over 5 per 1000. The growth in physician numbers in EU members states was also very variable; strong increases were observed in Austria, Spain, Sweden, and Denmark, while the number of doctors grew only modestly in France, Poland, and Slovakia. In most countries, the expressed concerns that governments are addressing relate primarily to shortages of general practitioners and doctors in rural and remote areas. Large-scale trends, such as population ageing and technological change are expected to continue to play a key role in increasing the demand for workers in healthcare, and most national projections foresee considerable growth of the employment needs in healthcare sectors, as is the case in the USA, Australia, and Canada.

### Increasing demand for imaging

Radiology in the EU has, to some extent, followed these trends by increasing numbers of radiologists, to a variable extent, depending on the country. Nonetheless, many countries are increasingly unable to cope with the growing demand for imaging procedures, which generally far outstrips any population-based changes in radiologist numbers over time. Increasing numbers of imaging examinations in recent decades are attributed to advances in technology (enabling fast and reliable imaging), to the ageing population (with more chronic diseases) and to the practice of defensive medicine (in part responsible for the fact that at least 20–30% of imaging procedures performed are not necessary and do not generate information that improves diagnosis or treatment, nor do they affect the patient’s health). UNSCEAR data have shown considerable annual growth in medical radiological examinations worldwide, with a 70% increase between 2000 and 2020 overall. In CT, the number of procedures and the collective dose have risen markedly between 2008 and 2020. The number of procedures has increased by about 80%, and the collective dose has increased by around 70% [21].

Although the number of radiologists has also been increasing over time, in many countries the huge demand for imaging has not been matched by a similar rate of increase in the radiologist density, deepening the gap between workforce availability and workforce requirement to deal with demands for imaging.

### Absolute numbers of radiologists per population (radiologist density)

As reported in Part 2 [3], the EU-REST main survey provided data on the numbers of radiologists in EU member states and their density. According to these data there are 60,771 radiologists in the EU-27, with an EU-wide ratio of 127 radiologists per 1,000,000 inhabitants (or 1.27 per 10,000 inhabitants). Bulgaria has the lowest number (51/million inhabitants) and Sweden the highest (270/million inhabitants).

### Ageing of the radiologist workforce

The ageing of the healthcare workforce is a particular concern, especially in countries in which a significant percentage of the workforce is aged 55 years and older. These countries face the imminent challenge of replacing retiring workers. A WHO report shows that 13 of 44 countries that reported data on this issue have a workforce in which 40% of medical doctors are aged 55 or older [20].

Considering those 17 countries that provided an age profile in the EU-REST survey, approximately 19% of their radiologists will retire in the next 5 years, and 45% are over 51 years old. Providing (1) a picture of retirement in 5 years (taking 66 years as a reference value, although the retirement age varies between countries) = potential immediate loss and (2) an overview of the impact considering the minimum age value of the 51–60 range = potential loss in the next 10 to 15 years would allow Member States and the EU as a whole to implement contingency measures.

The trend of ageing among the radiologist population is compatible with OECD data about medical doctors overall, where over one-third of doctors were above the age of 55 in 2019 (only 20% in 2000). The rapid ageing of all medical doctors is particularly visible in Italy, where the share of doctors above the age of 55 has increased from around 20% in 2000 to 56% in 2019, and in France where 14% of doctors in 2019 were over the age of 65. Nine EU member countries (Croatia, Czechia, Estonia, France, Hungary, Italy, Lithuania, Poland, Sweden) will lose over 20% of the radiologist workforce in the next 5 years due to retirement (considering the retirement age of 66 years), higher than the EU average (19%). Lithuania presents the highest value (35%).

Looking a few more years ahead, in Czechia, Estonia, France, Hungary, Italy, Lithuania and Sweden, more than 50% of the radiologists are over 51 years old, indicating a high demand for replacements in the next 15+ years.

### Full-time vs part-time working, and scope of practice

Absolute radiologist density does not account for the actual time that radiologists work to deliver services, and how this relates to numbers of full-time equivalents (FTEs). Neither does it account for the variations in services delivered (scope of practice). The scope of radiologists’ practice may differ widely within a single department and between different departments, depending on the type and quality of equipment used and type of radiological practice (US, CT, MRI, X-ray, diagnostic vs interventional radiology) performed, and the need for specific subspecialists. These variables must also be reflected in requirements for the specific subspecialised radiologist workforce. For instance, radiologists whose work encompasses interpretation of CT studies can read many more brain CTs per day than CTs of thorax-abdomen-pelvis. Radiologists performing US can perform many more thyroid US than complex Doppler examinations of peripheral arteries and veins.

Interventional radiologists performing complex procedures (e.g., EVARs, complex neurointerventions, complex hepatobiliary, urologic or vascular procedures) need different staffing levels compared to those performing mostly simpler interventions (drainages, biopsies, etc.).

### Distribution of radiologists

OECD data have shown considerable differences in the density of doctors between urban and rural areas, with, for example, huge differences in Hungary, Slovakia, Lithuania, and Latvia. In many countries, there is a particularly high concentration of doctors in national capital regions (Austria, Czechia, Greece, Hungary, Portugal, Slovakia). The same trends are present in radiology, and career opportunities and professional development are undoubtedly better in larger centres and academic institutions. Independent of staffing density guidelines, additional policies will be required in some countries to address this imbalance of distribution (e.g., providing financial incentives for radiologists serving in underserved areas, reorganising service delivery to improve working conditions of doctors working in more-remote areas, regulation of the choice of practice location for radiologists, etc.).

### Public vs private practice

ESR national society members report that radiology is an attractive profession in most EU states, and attracting young doctors to radiology does not seem to be the major problem. However, retaining radiologists in the public healthcare service is becoming difficult in many countries (notably, but not only, in France, Poland, Croatia, Slovenia), as private sector work often offers much higher income and a better work-life balance, while the majority of more-complex procedures and emergency services are performed in the public sector.

### Effects of the COVID-19 pandemic

The COVID-19 pandemic has accelerated or exacerbated many already existing problems that also affect retention of radiologists within healthcare services. Increased international mobility, coupled with higher income availability in richer countries, has led to a drain of radiologists from East to West within Europe. Working-from-home practices introduced during the pandemic out of necessity have become the norm in many circumstances, and have influenced radiologists’ willingness to work long hours, out-of-hours shifts, etc. Part-time practice has been increasingly embraced by radiologists (and many other groups within and outside healthcare services), arising from changed experience of work-life balance during the pandemic.

### Influence of artificial intelligence (AI)

Nobody can yet reliably predict how AI will affect radiology practice, but changes will obviously happen [22]. AI has great potential to optimise workflow in radiology, including improvement in referral of patients for radiology procedures and assistance to radiologists in interpretation of many studies. Many commentators have suggested that AI may therefore reduce the numbers of radiologists needed in the future.

Conversely, findings identified by incorporation of AI-enabled algorithms into radiology reporting pathways may actually substantially increase workload for radiologists, as many more findings may need to be specifically evaluated and investigated or dismissed. We will need radiologists who are skilled in the use of AI and digital health tools in general, but how AI will affect the daily workflow of radiologists is impossible to predict with certainty at present. Therefore, staffing guidelines need to be flexible and adaptive.

### Conclusion

While using crude population numbers as a denominator to determine the numbers of radiologists needed to provide services within a country has the apparent advantage of simplicity, this section has analysed and, we hope, demonstrated that such a crude method for determining a radiologist density guideline ignores many potentially confounding issues. Specifically, these include:

1. **The lack of any agreement on an appropriate radiologist density, given the very wide current variation among EU Member States**.
2. **The need to take account of changing demands for radiology services over time, independent of population numbers**.
3. **Varying age profiles of working radiologists among the EU 27**.
4. **Varying proportions of full-time and part-time work among radiologists**.
5. **Differing scopes of practices among institutions and countries**.
6. **The need to ensure equitable access to and distribution of radiologists for countries’ entire populations, not just those living in larger urban centres**.
7. **Varying proportions of public and private practice, and the influences these variations have on workforce retention**.
8. **Mobility of workforce and changing work practices, accelerated since the COVID-19 pandemic**.
9. **The as-yet unknown future influence of AI on radiologist work patterns**.

#### Based on workload

Again, this would seem, at first glance, to be a good basis for calculating workforce need. After all, a given amount of work should require a similar amount of time/effort in all EU Member States, assuming proper weighting could be determined and applied to different types of radiology work. However, even local attempts to use workload measures to determine workforce needs [5, 6, 10], have found large variability in how workload is calculated across different sites (often with a view to maximising apparent local/individual workload and/or income). If measurement of workload in radiology could be standardised, this could become a very effective denominator of staffing needs. However, such standardisation does not exist at present. We believe the development of a standard method for workload measurement at the European Commission level would be desirable, but doing so lies outside the scope of the EU-REST study and the EU’s SAMIRA (Strategic Agenda for Medical Ionising Radiation Applications) framework. However, if such standardisation could be achieved in the future, then the basis for radiologist workforce calculation could be adapted to take account of it.

An additional factor is that the evolution and maturity of preventive medicine within any country may influence the number of imaging studies done. For example, if a lung cancer screening programme exists in any given country, or if cardiac CT is readily available and incorporated within clinical practice, the numbers of CT studies done and of CT scanners and radiologists required will be higher than if these practices are not supported.

Other drawbacks of any crude workload-based calculation of radiologists need include (Table 2):

1. Regardless of the EU Working Time Directive [23], working conditions vary among countries and among centres within any one country. The number of days off per year, maximum working hours, etc., all can influence the total number of radiologists required to deliver a certain amount of workload.
2. Determining needed radiologist numbers on the basis of numbers of studies reported alone takes no account of the substantial proportion of modern radiology practice that does not necessarily result in a countable output, such as a radiology report. One of the most important aspects of many radiologists’ work is preparation for and participation in multidisciplinary team (MDT) meetings, with radiologists playing a significant direct role in decision-making for patient management. Additionally, patient advocacy groups are increasingly calling for direct access to radiologists to discuss their imaging [12, 16, 17, 24]. Such direct patient-radiologist engagement would be beneficial to all involved but would be ignored by workload measures based on the numbers of reports generated. Other aspects of radiologist work which are not easily counted by report numbers include interventional radiology (of variable complexity), supervision and teaching of trainees and other staff members, research, etc.
3. There is a huge inter-individual variation in reporting performance in terms of numbers of studies, independent of the maturity and experience of the reporting radiologist [25]. The speed at which individuals work cannot and should not be fixed or mandated; radiology is not a factory production line.
4. The availability of infrastructure (workstations, IT infrastructure, etc.) can influence reporting speed and productivity [24].
5. Organisational aspects of reporting environments can influence reporting efficiency (e.g., frequency of breaks during continuous periods of sustained concentration, frequency and numbers of interruptions, such as for phone calls, issues relating to patient management, protocol determination, justification, etc. [24]).

**Table 2:** Pros and cons: basing radiologist numbers on workload

Pros	Cons
Flexible to allow for differences in practices in different countries	Variability in how workload is counted between institutions
Adaptable for different institutions doing variably complex work	Difficulty incorporating some aspects of work (e.g., intervention, multidisciplinary work, patient consultation)
Allows for relatively rapid response to changing practices or new techniques	Liable to “gaming” to increase apparent workload
Faster changes in workforce recommendations facilitated, relative to population-based standard	Requires consistent verifiable data from institutions, with uniformity of counting method
	Requires very granular data to be accurate (e.g., not all CTs can be counted in the same way, CT brain takes much less time to interpret than multiphase body CT)
	Depends on specifics of clinical and public health practice within any country

#### Based on equipment or bed availability

Basing workforce needs on the number of pieces of relevant equipment or on the number of patient beds available in hospitals may be useful for some other medical specialists, but is less reliable as a basis for calculating radiologist needs.

With respect to hospital bed numbers, radiology is a specialty that provides services on both an in-patient and outpatient basis. Therefore, any attempt to link radiologist numbers to in-patient bed numbers would ignore outpatient work, which can, in some circumstances, form the majority of radiologists’ work. Additionally, in many developed countries, there is a substantial move towards managing many medical issues on a day-case or outpatient basis, which would once have required in-patient hospital admission, and also to speed up the discharge of patients from in-patient hospital beds after procedural treatment. Nonetheless, many patients managed in these ways will require imaging and/or interventional radiology services, which may continue after their discharge from an in-patient bed. Thus, radiology activity is in no useful way reflected by in-patient bed numbers.

With respect to equipment availability, tying radiologist numbers to numbers of CT or MR scanners, etc., takes no account of varying practices, complexity of medical work undertaken and utilisation of equipment. Usage of radiology equipment may be only during the normal 8–10 h working day, or around the clock (on a full-service or reduced activity basis), often depending on staff availability and/or demand for services. Reimbursement policies within different countries may influence equipment numbers. The efficiency of patient throughput through radiology equipment may also vary between institutions. The complexity of cases in an institution may have a major impact on throughput: CT or MR units dealing with seriously ill, immobile patients may perform fewer examinations than one dealing mostly with ambulatory patients with less-complex presentations, yet the radiologist time required to interpret studies on complex patients may be much greater.

On a broader level, the number of pieces of equipment will depend to some extent on the general structure of the healthcare system within each country. Depending on geography, transport infrastructure, population distribution, etc., imaging services may be widely distributed or concentrated within fewer, larger centres, and these factors will influence the total number of pieces of equipment needed to service the population (Table 3).

**Table 3:** Pros and cons: basing radiologist numbers on equipment or bed availability

Pros	Cons
Allows for differences between high-level and lower-level services	Ignores usage patterns of equipment (e.g., 24-h service, office hours only, etc.)
Allows for rapid changes in required workforce as equipment availability changes	Usage of in-patient beds varies hugely among countries, depending on the availability of beds, day-case access, and overall model of care
Faster changes in workforce recommendations facilitated, relative to population-based standard	Ignores variable efficiency in equipment utilisation (e.g., a department could be “rewarded” with additional staff by purchasing additional equipment, rather than utilising existing facilities more efficiently)
	Risks embedding inappropriately low equipping levels in a system, if availability is used at any particular point in time to determine necessary workforce.
	Does not automatically take into account greater time commitment for more-complex imaging studies

## ESR-proposed method to estimate and calculate radiologist workforce needs

### Proposed approach to staffing guidelines

Given the issues outlined in the sections above and to address the main challenge of establishing an adaptable, scalable guideline, we propose the use of **hour of machine/system/activity** as the basic unit.

Several advantages are expected from this approach:

- The idea behind this approach is to define the number of radiologists needed for each working hour of a certain type of radiological machine (i.e., MRI, CT, US, angiography (DSA), conventional radiology (X-ray), fluoroscopy, and others) or non-reporting activity (such as participation in a Multidisciplinary Team Conference), including reporting time, and non-reporting duties.
- By this, a basic unit would be introduced that could be used as the basis for the further calculation of staffing needs. This basic unit would be multiplied by the running hours for the specific imaging system or activity. Based on the working hours a radiologist is allowed/contracted to work per year in a certain country/institution, the number of required full-time equivalents can be calculated.

(In a publication by the Japanese College of Radiology, it was suggested to multiply the number of needed radiologists following a calculation as described above by a factor of 0.6, based on the assumption that about 60% of a radiologist’s working time is study reporting time [26]. An Irish review of measuring radiologist workload from 2011 estimated that approximately two-thirds of an average radiologist’s work time is taken up by countable activities, generating reports or performing procedures [6, 10]).

- This unit can also be used to calculate the need for additional workforce resulting from the planned expansion of an already existing modality/service or to calculate the need for workforce in case of an increasing number of systems. Conversely, if there is a shift from one type of examination towards another (for example, as observed in the past, away from diagnostic invasive angiography to non-invasive CT angiography), possible reductions of staffing needs could also be estimated based on such basic units.
- These units can also easily be adapted to possible specific needs and duties at certain institutions. For example, in academic or teaching institutions, units could be multiplied by a pre-determined factor to take account of specific supervision/teaching/research, etc., duties.
- In specific environments incorporating teaching/education, dedicated calculations can be provided to address the special circumstances. In the teaching setting, the time involvement of a fully trained, independently practising radiologist is reduced (especially after the first few weeks) since their permanent presence is not always needed throughout an entire shift (assuming that some work is done by trainees). It is expected that the trainee may, for example, be running a list (e.g., in CT, MRI) (we believe this to be a crucial part of the learning process) and the fully trained “teacher” joins in from time to time for case discussion, etc. However, the total workforce (fully trained and trainees) needed in the teaching situation is increased since trainees plus fully trained radiologists have to be assigned for each hour of service.
- These units can also easily be adapted according to changes in clinical practice. The availability of AI tools will certainly have an impact on the future work of radiologists, as discussed above. However, these tools may impact different fields and modalities more than others, and it remains unclear what the specific impact of AI availability will be. A basic unit, as proposed herein, should provide flexibility to adapt workforce numbers as the impacts of new developments become clear.

Following the hypothesis that examinations which are time-consuming in image acquisition are also time-consuming in analysis and interpretation, such a basic unit would also be rather independent of the varying case mix in different imaging centres. Similarly, the use of such a basic unit would be able to accommodate the needs for educational activities in academic centres, where the total number of examinations per hour might be lower than in centres which do not have an academic/educational function, based on the assumption of more-complex cases and possibly more time-consuming examinations. Based on such differences in practice, calculated units can also—as described above—easily be multiplied by a certain factor.

**Based on the abovementioned thoughts and assumptions, we propose the definition of one hour of system use as the basic unit, and we propose staffing requirements depending on modality as described in the following modality-specific sections**.

We describe the full reasoning and calculation process for Interventional Radiology (IR), with abbreviated comments for other modalities, followed by a worked example.

### Interventional radiology using digital subtraction angiography and fluoroscopy (IR)

#### Explanation of the procedures

Under this term/category, all vascular and non-vascular procedures performed under fluoroscopic guidance are included, under the general abbreviation IR.

The list of vascular procedures includes neurovascular procedures (endovascular treatment of ischaemic stroke, endovascular treatment of intracranial aneurysm, arteriovenous fistula and more), cardiovascular procedures (treatment of coronary artery disease, aortic disease, peripheral arterial disease, treatment of valvular diseases and much more) as well as embolisations in the non-oncological setting (bleeding embolisation) or in the oncological setting (tumour embolisation, radioembolisation, and more). Relatively newer procedures include endovascular treatment of acute pulmonary embolism by embolectomy and treatment of acute and chronic pelvic vein thrombosis.

The list of non-vascular procedures includes percutaneous biliary interventions, tumour ablations (microwave ablation, radiofrequency ablation, cryoablation, irreversible electroporation) as well as interventions in the intestinal and urogenital tract. Additionally, drainage, biopsies, and more are part of the spectrum of interventional radiology.

### Approach to staffing needs calculation

Beside the fact that all these procedures represent minimally invasive procedures performed under fluoroscopic guidance, all these procedures also have in common the fact that the performing physician has to be in the room for the entire procedure. There are few steps during most procedures that can be delegated, and no teleradiological approach is possible.

It has to be underlined that this estimation focuses on the work in the fluoroscopic suite. Additional clinical work at ward level (in some institutions, IR and Neuro-IR have their own beds) or in outpatient clinics is not included.

The room time of each patient ≠ the procedure time

Before each procedure can start, the patient must be prepared and the devices to be used must be selected and made ready. These preparatory steps do not always require the presence of the treating physician (interventional radiologist).

The time required for changeover of patients between cases differs between different institutions, depending on process organisation and the complexity of cases performed; this could influence the calculation of staff needed. However, during this changeover period, the interventional radiologist would typically write the report of the previous procedure and prepare for the next one, including communicating with other staff members about the planned upcoming procedure and the devices needed.

In smaller institutions without full-time interventional services, the interventionalist may be involved in other diagnostic work aside from interventional procedures (for example reporting CT cases). Thus, the running hours of an IR service itself cannot be used as a basis for workforce calculation.

The above-described work-up and preparation time depends of course on the complexity of the respective procedures. However, based on the assumption of a certain mix of complexity an estimation is done as detailed below:

The basic unit, as described above, refers to the room time of the patients.

**One hour IR (HR**_**IR**_**) as the basic unit to be used as the basis for staffing guidelines refers to 1** **h room time of the patients**.

### Proposed calculation (IR)

Based on the assumption above, the staffing recommendations are as follows:

**One hour IR (HR**_**IR**_**) requires 1.5 working hours of a board-certified interventional radiologist who is capable and licensed to work independently**.

### Working example

As a practical example: the placement of a transjugular portosystemic shunt (TIPSS) typically requires a procedure time of 60–120 min. The room time of the patient will be between 120 and 180 min. Consequently, the need for the interventionalist will be 3–4.5 h, to reflect the need for careful patient selection, communication/discussion with the referring physician, patient consent (ideally performed on the day before), checking the lab values, organising possible pre-treatment and writing the report. Additionally, time should remain available to check and organise the stock of devices needed.

If an IR service is running 5 days a week with 8 h patient room time a day, for 50 weeks per year, the total need would be to cover 2000 h per year. Doctors working 40 h per week, for 40 weeks a year (following the assumption of The Gishen Ready reckoner [27] to reserve 12 weeks for leave, study leave, illness, meetings, machine breakdown or non-function) are working 1600 h per year. Based on the estimation above, 3000 h should be covered.

Following this calculation, for an IR service being busy 5 days a week for 8 h, 1.875 IR specialists (effectively, 2) being able to work independently and unsupervised are required.

In the teaching setting, this demand on staff needed should be altered based on the need for continuous presence of a medical doctor being capable and licensed to work independently, to oversee all steps performed by a resident/fellow.

**One hour IR (HR**_**IR**_**) in the teaching situation requires 1.5 working hours of a board-certified interventional radiologist who is capable and licensed to work independently PLUS 1 working hour of a resident/fellow**.

#### Magnetic resonance tomography/imaging (MR)

MR examinations always consist of a carefully chosen and individualised combination of different techniques and sequences, which are selected based on the specific request and the clinical situation. The length of an MR examination depends on the number of sequences combined and, thus on the clinical scenario, as well as on the system used. The specific equipment determines the acquisition length of each sequence and shows huge variation.

In recent years, a significant reduction in the acquisition length has been observed thanks to technical developments, and there is an increasing trend towards short protocols, with the goals of optimising patient throughput and scanner use and increasing patient comfort (by reducing potentially uncomfortable time lying in the scanner). Consequently, the number of examinations/patients per working day/working hours tends to continuously increase. However, the reporting time needed per study has not changed. Increased scanner efficiency thus results in an increased need for radiologist workforce since the total workload for reporting and management is increased by the number of patients scanned.

**One hour MR (HR**_**MR**_**) is the basic unit to be used as the basis for staffing guidelines refers to 1** **h room time of the MR unit**.

**One hour MR (HR**_**MR**_**) requires 1.5 working hours of a board-certified radiologist who is capable and licensed to work independently**.

**One hour MR (HR**_**MR**_**) in the teaching situation requires 1.5 working hours of a Resident plus 1** **h of a board-certified radiologist who is capable and licensed to work independently**.

#### Computed tomography (CT)

CT examinations frequently involve careful decisions regarding the need to inject iodinated contrast material or not. The fear of nephrotoxic injuries caused by iodinated contrast materials (contrast-induced nephropathy, CIN) has decreased in recent years. Together with reduced iodine volumes needed for diagnostic purposes with modern CT scanners, there is a clear tendency towards broad use of contrast material in modern CT. Consequently, CT supervision by radiologists includes selecting the contrast administration and imaging phase protocol. Additionally, image reconstruction plays a substantial role in modern CT scanning, given the huge amount of image data acquired with very high spatial resolution. CT reporting without image post-processing is not appropriate nowadays. Whereas pure image acquisition in CT takes only a few seconds, the entire examination, including patient preparation, contrast administration, and post-processing, takes much longer. Furthermore, the difference in image acquisition time between a single body-part CT scan (for example, CT of the brain) and a body CT (chest, abdomen and pelvis for oncologic staging purposes, for example) is negligible (almost always below 1 min, other than in multiphase scanning involving deliberately delayed phases), but the difference in the post-processing and reporting time is much greater. Consequently, the acquisition/scan time cannot be used as a meaningful marker for workforce calculation in CT. In fact, the more advanced the scanner technology is, the shorter the acquisition time might be; but simultaneously, the higher the amount of data obtained will be, increasing post-processing and interpretation time.

Additionally, CT scanner improvements have led to new applications (e.g., CT brain perfusion) and reduced radiation dose, both of which have resulted in increased CT utilisation.

For all these reasons, even as actual CT scanning time per case reduces, radiologist time to interpret and report cases can actually increase, and the time for radiologists to cover the output of a functioning CT scanner can become longer.

**One hour CT (HR**_**CT**_**) is the basic unit to be used as the basis for staffing guidelines refers to 1** **h room time of the CT unit**.

**One hour CT (HR**_**CT**_**) requires 1.5 working hours of a board-certified radiologist who is capable and licensed to work independently**.

**One hour CT (HR**_**CT**_**) in the teaching situation requires 1.5 working hours of a Resident plus 1** **h of a board-certified radiologist who is capable and licensed to work independently**.

#### Interventional computed tomography (I-CT)

This category includes all diagnostic and therapeutic procedures performed using CT as image guidance, such as biopsies, drainages, ablations, etc. Depending on department organisation, this work may be incorporated into the duties of radiologists covering other IR or CT or may need to be considered separately.

**One hour I-CT (HR**_**I-CT**_**) is the basic unit to be used as the basis for staffing guidelines refers to 1** **h room time of the patients**.

**One hour I-CT (HR**_**I-CT**_**) requires 1.5 working hours of a board-certified interventional radiologist who is capable and licensed to work independently**.

**One hour I-CT (HR**_**I-CT**_**) in the teaching situation requires 1.5 working hours of a board-certified interventional radiologist who is capable and licensed to work independently PLUS 1.5 working hours of a resident/fellow**.

#### Positron emission tomography (PET) (hybrid imaging)

This category includes hybrid imaging (PET/CT and PET/MR) when this work forms part of the activity of a radiology department and is performed/reported by radiologists.

Despite improvements and innovations in PET scanner technology that have led to improved spatial resolution, faster acquisition and higher detector sensitivity (which can in turn be used to decrease the injected radiotracer dose and, therefore, radiation exposure), major factors such as post-injection delay have remained unaffected by these technical developments. While no data on average reading times exist for PET, reading times can be expected to be approximately 30 min, given that (1) PET scans are practically always whole-body (or even total body, head-to-toe) scans; the vast majority are oncologic cases, frequently of a high complexity level, and (2) both uptake measurements on PET (standardised uptake values, SUV) and lesion size measurements on the CT component need to be included in the report. In addition, as for CT, the reporting time is just one part of a radiologist’s duty; others include: establishing intravenous access, radiotracer injection, and interaction with patients and referring physicians, as well as with technicians about study indication, the scan protocol (anatomic range, contrast, and full/low-dose CT).

**One hour PET as the basic unit to be used as the basis for staffing guidelines refers to 1** **h room time of the PET unit**.

**One hour PET requires 1.5 working hours of a board-certified PET-trained radiologist/CT-trained Nuclear Medicine Physician who is capable and licensed to work independently**.

**One hour PET in the teaching situation requires 1.5 working hours of a Resident plus 1** **h of a board-certified PET-trained radiologist/CT-trained Nuclear Medicine Physician who is capable and licensed to work independently**.

#### Plain X-ray (XR)

These examinations are typically performed independently of direct supervision by the radiologist in charge, are highly standardised and usually do not require direct pre-acquisition interaction with radiologists. The reporting of these examinations is independent of the image acquisition, and interruptions due to acute requests or questions about indication and imaging technique are significantly less frequent when compared to other modalities (CT, MR).

**One hour XR (HR**_**XR**_**) as the basic unit to be used as the basis for staffing guidelines refers to 1** **h running time of the respective X-Ray unit**.

**One hour XR (HR**_**XR**_**) requires 0.5 working hours of a board-certified radiologist who is capable and licensed to work independently**.

**One hour XR (HR**_**XR**_**) in the teaching situation requires 0.5 working hours of a board-certified radiologist who is capable and endorsed to work independently PLUS 0.5 working hour of a resident/fellow**.

#### Fluoroscopy (Fluoro)

This category comprises all non-interventional (i.e., not requiring percutaneous access) examinations performed under fluoroscopy (e.g., gastrointestinal or urogenital contrast studies). The specific characteristic of these examinations can be described by their dynamic character; the assessment and consequently diagnosis is usually made “on the fly”, during the fluoroscopic examination. Thus, the continuous presence of the person in charge is required during the entire examination. Reporting of these examinations typically takes place during subsequent examinations (and is included in the calculation).

**One hour Fluoro (HR**_**Fluoro**_**) as the basic unit to be used as the basis for staffing guidelines refers to 1** **h time of patient service**.

**One hour Fluoro (HR**_**Fluoro**_**) requires 1.0 working hour of a board-certified radiologist who is capable and licensed to work independently**.

**One hour Fluoro (HR**_**Fluoro**_**) in the teaching situation requires 1.0 working hours of a board-certified radiologist who is capable and endorsed to work independently PLUS 1 working hour of a resident/fellow**.

#### Sonography/ultrasound/duplex/Doppler-ultrasound (Sono)

The specific characteristic of these examinations can be described by their dynamic character; the assessment and consequently the diagnosis is usually made “on the fly”, during the US examination. The continuous presence of the person in charge is required during the entire examination. In some countries/centres, selected US examinations are performed under standardised conditions by specially trained staff (Technicians, Radiographers, Sonographers). However, many examination types are performed by radiologists only, and in many countries, there are no sonographers. Consequently, US is (and will remain) one of the central basic modalities in radiology and remains a central duty in most radiologist’s clinical routine. Our calculations and recommendations are based on the presumption that US is performed by radiologists. Even if the scanning for some studies is performed by sonographers, the images must be directly viewed, and the reports must be produced by radiologists. Reporting of completed US examinations takes place during subsequent examinations (and is included in the calculation).

**One hour Sono (HR**_**Sono**_**) as the basic unit to be used as the basis for staffing guidelines refers to 1** **h time of patient service**.

**One hour Sono (HR**_**Sono**_**) requires 1.0 working hour of a board-certified radiologist who is capable and licensed to work independently**.

**One hour Sono (HR**_**Sono**_**) in the teaching situation requires 0.5 working hours of a board-certified radiologist who is capable and licensed to work independently PLUS 1 working hour of a resident/fellow**.

#### Multidisciplinary team (MDT) meeting

Multidisciplinary teams (MDT) are created in clinical medicine to bring together a group of healthcare professionals from different specialties in order to agree on diagnoses and determine patients’ treatment plans. One important function of such MDTs is to meet regularly to have an interdisciplinary discussion to optimise patient-centred medical care. Initiated in oncologic medicine, MDTs have also become established in many fields of clinical medicine, e.g., cardiology, vascular surgery/medicine, epilepsy care, inflammatory bowel disease, paediatrics, etc.

As a consequence of the continuous increase in the technical capabilities and diagnostic accuracy of modern imaging, radiology is a central part of most such MDTs, and many MDT meetings take place in radiology departments with active participation of (and frequently chairing by) radiologists.

With increasing specialisation in modern clinical medicine, the number of MDTs is continuously increasing, and the request for regular MDT meetings represents a disruptive change in the daily routine in clinical Radiological departments. The most time-consuming activity is the preparation of such MDT meetings. Given the fact that decisions of the highest importance for future patient care are made in such meetings, careful preparation and assessment of ALL available imaging data are required and expected from the participating radiologist. When calculating/estimating the workforce needed to cover such MDT meetings, the preparation time should be included, and a 2:1 approach (2 h preparation for each 1 h of MDT activity) is realistic.

**One hour MDT (HR**_**MDT**_**) as the basic unit to be used as the basis for staffing guidelines refers to 1** **h MDT-meeting time**.

**One hour MDT (HR**_**MDT**_**) requires 3 working hours (i.e., 2** **h preparation and 1** **h of meeting conduct) of a board-certified radiologist who is capable and licensed to work independently**.

While minor adaptations to local situations might be required in certain cases, the aim is to offer a single formula that is applicable in all EU-27 countries. Referring to 1 h machine time as the basic unit should facilitate adaptation to local situations by slightly changing the conversion factor between machine hours and working hours for Radiologists.

For all modalities, these estimations and assumptions are dedicated to routine service provision during normal working hours. There is an enormous variety of on-call organisations among different countries, cities, and institutions. For these on-call and/or out-of-hour services, different calculations are needed, indicating additional staffing needs. Nonetheless, the formula is adaptable to on-call services.

Future adaptations of this formula, in response to changes in technology and practice patterns, could be easily achieved by agreed (and approved by the relevant authorities) adaptations to the multipliers and weighting factors contained therein without requiring extensive effort to establish new principles.

**Worked Example**

Table 4 shows a worked example for a typical, relatively small hospital department, where all modalities are in use for 8 h per day, 5 days per week, 50 weeks per year (allowing for public holidays, etc.). In this department, 4 MDT meetings per week are conducted with radiologist involvement. No radiologists in training participate in work in this department, and therefore training needs are not included in calculations.

**Table 4:** Example of staffing need calculation

Modality	No. of units	Working hours per day	Working days per week	Working hours per year	MDT hours per week	Conversion factor	Radiologist hours needed per year	Radiologist hours available per year per person	No. of radiologists needed
			50 weeks						
IR	1	8	5	2000		1.5	3000	1600	1.875
MR	2	8	5	2000		1.5	6000	1600	3.75
CT	2	8	5	2000		1.5	6000	1600	3.75
PET/CT	1	8	5	2000		1.5	3000	1600	1.875
Plain radiographs	2	8	5	2000		0.5	2000	1600	1.25
Fluoroscopy	1	8	5	2000		1	2000	1600	1.25
US	4	8	5	2000		1	8000	1600	5
MDTs					4	3	624	1600	0.39
Total				14,000			31,824		19.14

A spreadsheet is available in the Electronic Supplementary Material, which can be used to apply these formulae to any radiology department. Options are available for departments without and with trainees, with the relevant conversion factors embedded in the two available worksheets.

## Education and training guidelines for radiologists

The main goal of these guidelines is to establish harmonised training requirements regarding duration and content for radiology training (residency) programmes within Europe in order to increase mobility and comparability.

### Recommendations for the education and training of radiologists

1. **Harmonisation of duration and content of training within the EU member countries**

In order to facilitate free mobility between member states of the European Union and to enhance the quality of radiological care for patients, harmonisation of education and training of Radiologists is desirable. Based on the data described previously [2], obtained from the survey performed as part of the EU-REST study, and on existing guidelines, there is still some variation in the length of radiology specialty training among EU Member States. A specialty training programme lasting 5 years, however, has already become a generally accepted European standard and should be established in all countries (additional time can thereafter be spent on further subspecialty training—see below). The EU Professional Qualifications Directive [28], which still recommends a minimum training period of 4 years, should be adapted accordingly. Most EU Member States already have a radiology specialty training duration of 5 years or longer (see Table 4 of Part 2 [3]). For those which currently have shorter durations of 4 or 4.5 years, a gradual transition, supported by a common curriculum (the recommended ETC—see below) should be encouraged.

The European Society of Radiology (ESR), as the key transnational provider of radiological education within Europe, has defined the content, structure, and duration of the specialty training programme in radiology. Initially introduced as the European Training Charter in Radiology, the European Training Curriculum (ETC) [29] provides a clear recommendation for a modern, structured training programme in Radiology. The content was defined in close cooperation with the relevant radiology subspecialty societies (Breast, Cardiovascular, Interventional, Musculoskeletal, Chest, Neuroradiology, Head-Neck, Paediatric, Gastrointestinal, Urogenital, Gynaecological, Emergency), and it is structured according to the required knowledge, skills, competencies, and attitudes. This ETC is continuously updated and represents an ideal blueprint for harmonised radiology education in Europe.

This ETC is supported by 38 National Radiology Societies, but not all these countries have yet implemented this ETC as the basis for their training programmes.

**Recommendation:**

- **Establish 5 years as the standard duration of specialty training in radiology**
- **Establish the ETC (in its continuously updated form) as a European-wide standard for radiology education and training**.

2. **Harmonisation of training structure within the EU member countries**

The ETC differentiates between levels I (first 3 years of training), II (years 4–5) and III (Fellowship—subspecialisation). This structure differentiates between basic general education and training in years 1–3 and more advanced training, usually in selected subspecialty fields in years 4–5. After successful completion of these 5 years, training to become a “General Radiologist” is finished. This basic 5-year structure is applied in many countries. Further harmonisation is desirable.

However, regarding Level III education, universally accepted European standards are missing. Structured Fellowship programmes are established in a few countries, and their duration varies between 1 and 2 years. In some countries, dedicated subspecialty training is provided in selected fields (e.g., neuroradiology, interventional radiology, paediatric radiology), but even in these fields, a common European standard is not accepted. Establishing formal subspecialty training requires political decisions and funding in most countries. Most European subspecialty radiology societies have produced training curricula for training in their respective subspecialties; as with the ETC-based standardisation recommended above, these (combined with ETC Level lll curricula) could and should be adopted as standard bases for formal radiology subspecialty training.

**Recommendation:**

- **Establish coordinated and standardised Fellowship programmes after the end of the general radiology 5-year residency training. Such Fellowships should generally last 1 year. Curricula for training in radiology subspecialties should be based on a combination of ETC Level III and specific subspecialty society-sponsored curricula**.

Training and education in radiology require a mix of knowledge and competencies; volume-based competency is one factor in supporting quality. Currently, the ETC does not define hours of teaching (European Credit Transfer System (ECTS))/education per field, numbers of cases to be reported or numbers of procedures to be performed. Training programme outlines in many countries do include such numerical recommendations. A definition based on case numbers is an insufficient parameter on its own to determine competence, and the useful threshold between granular measurement (to ensure a realistic and beneficial case mix) and applicability is difficult to establish. Nonetheless, it seems self-evident that it would be helpful to define a minimum number of cases/procedures to be reported/performed in each subspecialty.

Additionally, a minimum of required ECTS in each subspecialty should be defined.

**Recommendation:**

- **Establish a minimum requirement for a combination of ECTS and case/procedure numbers for each subspecialty, based on the ETC. This should be used in all EU member countries**.

Consequently, education in radiation protection, patient safety and quality control should be standardised following the same principles as above. Each fully trained radiologist should be qualified and well-educated in these essential fields. In most countries, radiation protection, safety, and quality management are established in the training programme, but the number of hours of teaching (ECTS) and the extent of practical training are not specified. Specific training in Radiation Protection should be organised following the recently published guidelines (EC RP 175 [30]).

**Recommendation:**

- **Establish a minimum requirement for a combination of ECTS and practical training in radiation protection, safety and quality management within the ETC. This should be used in all EU member countries**.

3. **Harmonisation of certification of completion of training within EU member countries**

In many, but not all, EU member countries, completion of specialty training is marked by formal certification. In many countries, this is based on a structured formal examination. In other countries, completion of training is determined as a dialogue among colleagues, and in some countries training completion is determined by spending a period of defined time in training (3–5 years).

The European Board of Radiology (EBR) has established the European Diploma in Radiology (EDiR), achieved by success in a formal standardised examination taken after completion of formal time-based training; this diploma is fully endorsed by the European Union of Medical Specialists (UEMS) and ESR.

As indicated on the website of the EDiR [31], “the EDiR is recognised as equivalent to: the exit training examination in Poland and in the Netherlands, the Croatian National Board examination, the image interpretation part of the Finnish national examination and the Flemish Board Examination in Belgium”.

Moreover, the EDiR has significant value in many other countries, such as France, Italy, Belgium, Sweden, Russia, Bosnia and Herzegovina, Slovakia, Malta, Estonia, and Georgia, where EDiR holders can use the certificate for professional credentialing and classification purposes. This is also the case in most countries in the Middle East and Asia, especially in India and Pakistan, where the EBR has special agreements with the corresponding national radiology associations [28].

**Recommendations:**

- **Formally complete training in radiology by administration of a harmonised and standardised examination to all trainees, in all European countries.**
- **Promote acceptance of the EDiR as equivalent to the national or specialty examination in radiology or—in countries without such specialty examination—to establish the EDiR as a requirement for certification of completion of training.**
- **In those countries which already have established examinations which must be passed to complete training, local evaluation of equivalence with the EDiR may be helpful to ensure harmonisation of standards.**

4. **Clear acknowledgement of trainees in workforce calculation**

As described above, education in Radiology is mainly a combination of practical and clinical education. Acquiring volume-based competency is a central part of Radiology training. There is a clear correlation between case load and experience. With increasing time within training, independent work of the trainees becomes more valuable to patient care and represents a central part of training at Level II (years 4–5).

As elaborated in greater detail in the guidelines for staffing detailed above, trainees must be taken into account while calculating workforce needs. Teaching is time-consuming (on the part of the teacher); conversely, trainees can deal with some parts of routine work and can contribute positively to department outputs. With increasing trainee experience, less time investment by the teacher is required. In the interventional setting, however, continuous presence of the fully qualified radiologist (teacher) is needed.

As a consequence, we proposed in the Guidelines for Radiologist Workforce (above) modality-depended modifications of the staffing calculation in the educational/teaching setting.

**Recommendation:**

- **Take account of the needs of trainees, and their contribution, in calculating workforce requirements, by incorporation in formulae (as outlined in the radiologist staffing guidelines)**

5. **Harmonisation of training centre evaluation within EU member countries**

In order to provide a harmonised quality assessment of training programmes and to ensure common high standards, the European Training Assessment Programme (ETAP) was introduced some years ago and was updated by the ETAP 2.0 programme.

ETAP is a joint initiative of the EBR and the UEMS Section of Radiology.

This programme represents a formal quality assessment for radiology training programmes. It is in line with the ESR European Training Curriculum (ESR ETC) [28].

According to the ETAP description, this certification “allows to check the level of competence, attitude and development of new skills that trainees acquire during the training period.” Furthermore, it provides an objective assessment of training departments and serves as an indicator and benchmark for training departments among trainees.

**Recommendation:**

- **Establish the ETAP certificate as a prerequisite for training centre accreditation in Europe**.

6. **Harmonisation of continuing professional development**

The Accreditation Council in Imaging (ACI), together with the UEMS, has established criteria for accreditation of educational events. Different rules and regulations apply for live education events (LEE), e-learning material, and blended learning and webinars. For certain amounts of educational activities, Continuing Medical Education (CME) credits can be claimed. This concept provides a very high level of standardisation. As a consequence of a very fruitful collaboration between the UEMS and ACI, the roles are clearly defined: ACI is responsible for assessing the content of educational events, and UEMS defines the rules and provides the credits.

However, in some countries, local CME credits are provided following different rules and regulations, and direct exchange and acceptance of UEMS CME credits (EACCME) is not possible among all countries.

**Recommendations:**

- **Establish the EACCME as the European currency for CME credits, and accept these credits in all countries as proof for continuous medical education**.
- **Establish a minimum number of CME credits which need to be obtained in a defined period of time to prove continuous medical education, and use this number in all European countries**.

#### Education and training recommendations for radiologists

- Establish 5 years as the standard duration of specialty training in radiology
- Establish the ETC (in its continuously updated form) as a European-wide standard for radiology education and training.
- Establish coordinated and standardised Fellowship programmes after the end of the general radiology 5-year residency training. Such Fellowships should generally last 1 year. Curricula for training in radiology subspecialties should be based on a combination of ETC Level III and specific subspecialty society-sponsored curricula.
- Establish a minimum requirement for a combination of ECTS and case/procedure numbers for each subspecialty, based on the ETC. This should be used in all EU member countries.
- Establish a minimum requirement for a combination of ECTS and practical training in radiation protection, safety and quality management within the ETC. This should be used in all EU member countries.
- Formally complete training in radiology by administration of a harmonised and standardised examination to all trainees, in all European countries.
- Promote acceptance of the EDiR as equivalent to the national or specialty examination in radiology or—in countries without such specialty examination—to establish the EDiR as a requirement for certification of completion of training.
- In those countries which already have established examinations which must be passed to complete training, local evaluation of equivalence with the EDiR may be helpful to ensure harmonisation of standards.
- Take account of the needs of trainees, and their contribution, in calculating workforce requirements, by incorporation in formulae (as outlined in the radiologist staffing guidelines)
- Establish the ETAP certificate as a prerequisite for training centre accreditation in Europe.
- Establish the EACCME as the European currency for CME credits, and accept these credits in all countries as proof for continuous medical education.
- Establish a minimum number of CME credits which need to be obtained in a defined period of time to prove continuous medical education and use this number in all European countries.

## General recommendations for all professional groups

These comprise recommendations which are applicable to all professional groups covered by the EU-REST project.

1. **National Registries**

A. Due to the abovementioned general lack of existing metrics about workforce availability for all relevant professional groups, and an absence of any widely applicable future-proofed standards for appropriate staffing levels. It is recommended that **each EU Member State should maintain a central registry for each professional group, and for equipment relevant to the performance of their work. Each Member State should ensure (ideally uniform) high quality of the data, including information on the**
  Number of professionals (and, if possible, number of whole-time equivalents)
  Age and gender profile of professionals (to allow for planning of training positions for future staff, retirement replacements, etc.)
  Appropriate qualifications needed for inclusion in the registry, and for licensing for independent practice
B. Such registries should, ideally, operate on common standards across all EU Member States, to ensure a meaningful cross-comparison of data. To provide for this, the definitions used to collate and verify the data contained within these registries should be common for all Member States. Data maintained in such registries should be shared through the EC, to facilitate the collation and maintenance of EU-wide data. The establishment of national registries (as outlined in 1A above) should be undertaken immediately, with harmonisation of standards and definitions and sharing of data across the EU to follow subsequently, once practical experience in establishment and maintenance of registries has been accumulated in Member States.

2. **Continuing professional development (CPD)**
  CPD in radiation protection is already required under the Basic Safety Standards Directive (BSSD), which has been transposed into national law in each Member State.
  **Mandated CPD should also include techniques and knowledge relevant to each professional group, beyond radiation protection issues**. The exact methodology and requirements for CPD for each group is a matter for each Member State, but adoption of the general principle of its being mandated should be accepted by each state.
3. **Adoption vs adaptation of guidelines**
  The clear recommendation from the EU-REST consortium is that each Member State should adopt the recommendations, which will encourage uniformity of standards and practice and, thereby, ultimately improve patient safety. If adoption of the guidelines is not possible in certain settings for justified reasons, relevant countries might adapt the proposed guidelines to make them applicable in their national context. The extent of such adaptation should, however, be limited.
  Fundamentally, consortium members believe that **adoption of recommendations by all Member States in a uniform manner would likely be more beneficial than adaptation of the recommendations. Full adoption should be the goal of the study and of the European Commission**.
4. **Harmonisation of training**

For each professional group, **harmonisation of training across all 27 EU Member States** (in terms of duration, curriculum, and certification of successful completion) is desirable and should be supported. This would benefit the interchangeability of qualifications across Member States and facilitate the mobility of relevant professionals. Specifics regarding this are given above for radiologists.

## Conclusion

The EU-REST project has revealed wide variation among the 27 EU Member States in terms of radiologist workforce availability, and education/training standards and duration. Similar findings apply to the other professional groups included in the project. While some progress is being made towards increasing the standardisation of training duration and curricula for radiologists, harmonisation can and should be accelerated, following the clear recommendations given above. This will ensure that professional standards are similar across the European Union, enhancing patient care, and providing for greater and easier professional mobility.

Workforce numbers are hugely variable across the EU 27, reflecting different models of service delivery, funding and resourcing, and historical healthcare system development. Extensive searching revealed no usable standard which could meaningfully be applied transnationally to reduce this variability, or even to define appropriate staffing levels. The EU-REST consortium has, accordingly, developed a new standard set of formulae which can be used and adapted for any modality and for varying types of radiology practice to determine appropriate radiologist staffing numbers to safely and competently deliver services. These formulae have been accepted by the European Commission as part of the final project report as appropriate radiologist staffing standards [32]. They can be simply used in any practice setting to determine the required workforce, and we hope they will become the standard against which staffing levels across the EU 27 will be measured, compared and harmonised in the future.

As explained in Part 1 of this series [2], one aim of the SAMIRA action plan is to “improve workforce availability, education and training aiming to mitigate the gaps between workforce supply and demand and ensure that all categories of staff involved in radiology, radiotherapy and nuclear medicine receive adequate education, training and continuous professional development in quality and safety issues”. Furthermore, the EU-REST project was intended to “address the needs for highly qualified workforce and proper forecasts of staff”. We hope that the outcomes of this project, and the guidelines and recommendations which derive from it (now accepted and published by the European Commission [32]), will help to advance this agenda, by guiding national healthcare systems in EU Member States towards harmonisation of standards, and mobility and interchangeability of personnel.

## Supplementary information

Supplementary Data

## Data Availability

European Commission: European Health and Digital Executive Agency, Analysis on workforce availability, education and training needs for the quality and safety of medical applications involving ionising radiation in the EU—Status and recommendations—Final report, Publications Office of the European Union, 2025, https://data.europa.eu/doi/10.2925/2213975.

## Abbreviations

EC: European Commission
ESR: European Society of Radiology
EU: European Union
EU-REST: European Union Radiation, Education, Staffing & Training
HaDEA: European Health and Digital Executive Agency
SAMIRA: Strategic Agenda for Medical Ionising Radiation Applications
UEMS: European Union of Medical Specialists
WHO: World Health Organization
