# Correction: Guidelines and recommendations for radiologist staffing, education and training

**DOI:** 10.1186/s13244-025-01982-y

**Published:** 2025-05-15

**Authors:** Adrian P. Brady, Christian Loewe, Boris Brkljacic, Graciano Paulo, Martina Szucsich, Monika Hierath, on behalf of the European Society of Radiology

**Affiliations:** 1 Department of Radiology, University College Cork, Cork, Ireland; 2 Division of Cardiovascular and Interventional Radiology, Department of Biomedical Imaging and Image-Guided Therapy, Medical University of Vienna, Vienna, Austria; 3 Department of Diagnostic and Interventional Radiology, University Hospital Dubrava, University of Zagreb School of Medicine, Zagreb, Croatia; 4 Polytechnic University of Coimbra, IPC-ESTESC—Coimbra Health School, Medical Imaging and Radiotherapy, Coimbra, Portugal; 5 H&TRC—Centro de Investigação em Saúde e Tecnologia, Lisbon, Portugal; 6 European Society of Radiology, Vienna, Austria

**Correction to:** ***Insights into Imaging*** (2025) 16:57;

10.1186/s13244-025-01926-6; published online 12 March 2025

In Table 4 of this article the value in row “IR” column “Radiologist hours needed per year” was incorrectly given as 300 but should have been 3000.

Table 4 with the incorrect value can be seen below:

**Table 4:** Example of staffing need calculation

Modality	No. of units	Working hours per day	Working days per week	Working hours per year	MDT hours per week	Conversion factor	Radiologist hours needed per year	Radiologist hours available per year per person	No. of radiologists needed
			50 weeks						
IR	1	8	5	2000		1.5	300	1600	1.875
MR	2	8	5	2000		1.5	6000	1600	3.75
CT	2	8	5	2000		1.5	6000	1600	3.75
PET/CT	1	8	5	2000		1.5	3000	1600	1.875
Plain radiographs	2	8	5	2000		0.5	2000	1600	1.25
Fluoroscopy	1	8	5	2000		1	2000	1600	1.25
US	4	8	5	2000		1	8000	1600	5
MDTs					4	3	624	1600	0.39
Total				14,000			31,824		19.14

Corrected Table 4 is shown below:

**Table 4:** Example of staffing need calculation

Modality	No. of units	Working hours per day	Working days per week	Working hours per year	MDT hours per week	Conversion factor	Radiologist hours needed per year	Radiologist hours available per year per person	No. of radiologists needed
			50 weeks						
IR	1	8	5	2000		1.5	3000	1600	1.875
MR	2	8	5	2000		1.5	6000	1600	3.75
CT	2	8	5	2000		1.5	6000	1600	3.75
PET/CT	1	8	5	2000		1.5	3000	1600	1.875
Plain radiographs	2	8	5	2000		0.5	2000	1600	1.25
Fluoroscopy	1	8	5	2000		1	2000	1600	1.25
US	4	8	5	2000		1	8000	1600	5
MDTs					4	3	624	1600	0.39
Total				14,000			31,824		19.14

